# Human ethmoid sinus mucosa: a promising novel tissue source of mesenchymal progenitor cells

**DOI:** 10.1186/scrt404

**Published:** 2014-01-24

**Authors:** Kyu-Sup Cho, Hee-Young Park, Hwan-Jung Roh, Dawn T Bravo, Peter H Hwang, Jayakar V Nayak

**Affiliations:** 1 Department of Otorhinolaryngology and Biomedical Research Institute, Pusan National University School of Medicine, 1-10 Ami-dong, Seo-gu, Busan 602-739, Korea; 2 Department of Otorhinolaryngology and Research Institute for Convergence of Biomedical Science and Technology, Pusan National University Yangsan Hospital, Yangsan, South Korea; 3 Department of Otolaryngology-Head and Neck Surgery, Stanford University School of Medicine, 300 Pasteur Drive, Edwards Building, R113, Stanford, CA 94305, USA

## Abstract

**Introduction:**

The identification of new progenitor cell sources is important for cell-based tissue engineering strategies, understanding regional tissue regeneration, and modulating local microenvironments and immune response. However, there are no reports that describe the identification and isolation of mesenchymal progenitor cells (MPCs) from paranasal sinus mucosa, and compare the properties of MPCs between tissue sources within the sinonasal cavity. We report here the identification of MPCs in the maxillary sinus (MS) and ethmoid sinus (ES). Furthermore, we contrast these MPCs in the same individuals with MPCs from two additional head and neck tissue sources of the inferior turbinate (IT) and tonsil (T).

**Methods:**

These four MPC sources were exhaustively compared for morphology, colony-forming potential, proliferation capability, immunophenotype, multilineage differentiation potential, and ability to produce soluble factors.

**Results:**

MS-, ES, IT-, and T-MPCs showed similar morphologies and surface phenotypes, as well as adipogenic, osteogenic, and chondrogenic differentiation capacity by immunohistochemistry and qRT-PCR for defined lineage-specific genes. However, we noted that the colony-forming potential and proliferation capability of ES-MPCs were distinctly higher than other MPCs. All MPCs constitutively, or upon stimulation, secrete large amounts of IL-6, IL-8, IL-10, IFN-γ, and TGF-β. After stimulation with TNF-α and IFN-γ, ES-MPCs notably demonstrated significantly higher secretion of IL-6 and IL-10 than other MPCs.

**Conclusions:**

ES-MPCs may be a uniquely promising source of MPCs due to their high proliferation ability and superior capacity toward secretion of immunomodulatory cytokines.

## Introduction

Mesenchymal progenitor cells (MPCs) represent an important progenitor cell population with multipotent capabilities which may have high utility for translational clinical applications. MPCs can be isolated from bone marrow (BM-MPCs) and differentiate into several mesenchymal lineages both *in vitro and in vivo*, such as bone [1-3], cartilage [1,2], adipose tissue [1,2] and muscle [1,4]. In addition to their multilineage potential, MPCs have been shown to possess immunomodulatory properties with therapeutic potential to prevent graft-versus-host disease (GVHD) in allogeneic hematopoietic cell transplantation [5]. Given these properties, MPCs have emerged as a promising tool for therapeutic applications in tissue engineering and regenerative medicine [6,7].

Although the BM has been the main source for the isolation of multipotent MPCs, BM procurement has numerous potential downsides, including pain, donor site morbidity and poor cell yield upon harvest. In the search for more optimal donor site substitutes, MPCs have been isolated from a number of adult tissues, including adipose tissue [8], synovial membrane [9], muscle [10,11], dermis [11], skin [12], trabecular bone [13], thymus [14], salivary gland [15], palatine tonsil [16] and, most recently, nasal mucosa [17].

The paranasal sinuses are a group of four paired air-filled cavities of the upper respiratory system that surround the central nasal airway. Two of these sinuses, the maxillary sinus (MS) and ethmoid sinus (ES), are both present at birth, and are the most common sites of sinonasal inflammation in both pediatric and adult populations [18]. The mucosa of both the MS and ES is an underappreciated source of donor tissue that is readily accessible to any ear, nose and throat surgeon during endoscopic sinus surgery. As many superficial undiseased mucosal sites of the sinonasal tract still require dissection to provide patency to the deep-seated sinuses, this fairly abundant ‘bystander’ regional tissue can be uneventfully harvested from patients in a minimally invasive, virtually painless, low morbidity manner. Although recent studies suggest the presence of MPCs in the mucosa of the inferior turbinate (IT) within the nasal cavity, and osteoprogenitor cells in the MS mucosa [17,19,20], there are no reports identifying and isolating MPCs from the paranasal sinus mucosa or comparing the properties of MPCs by tissue source within the sinonasal cavity. The purpose of this study was to assess the presence and properties of MPCs in human MS and ES mucosa and to compare MPCs isolated from four different sources (MS, ES, IT and tonsil) in the same individuals with respect to their morphology, proliferation capacity, colony forming capacity, immunophenotype, ability to generate soluble regulatory cytokines and potential for multilineage differentiation.

## Materials and methods

### Isolation and culture of MPCs

After obtaining Institutional Review Board (IRB) approval of Pusan National University Hospital, healthy, uninfected mucosal tissue specimens from the MS, ES and IT were obtained from adult patients undergoing endoscopic sinus surgery for chronic rhinosinusitis. Thirteen patients (eight males, five females) were enrolled from ages 20 to 56 years, (mean = 35.7 years) who each provided informed consent under an IRB-approved protocol. Tonsil (T) tissues were obtained after informed consent from four of these patients undergoing both endoscopic sinus surgery for chronic rhinosinusitis and tonsillectomy for obstructive sleep apnea. Tonsils served as a control tissue population for this study. All specimens were completely normal macroscopically and histology showed no evidence of inflammation in the part of the specimen that was obtained.

To isolate IT, MS, ES and T-derived MPCs, mucosa and tonsil tissues were washed extensively with an equal volume of phosphate-buffered saline (PBS) in order to remove the majority of erythrocytes. Tissues were cut into 1 to 2 mm pieces, and digested with 0.075% collagenase type I (Sigma, St. Louis, MO, USA) at 37°C for 30 minutes. Enzyme activity was neutralized with α-modified Eagle’s medium (α-MEM) containing 10% fetal bovine serum (FBS) and the sample was centrifuged at 1,200 × g for 10 minutes. The cell pellet was filtered through a 100 μm nylon mesh to remove cellular debris and adhered overnight at 37°C/5% CO_2_ in control medium (α-MEM, 10% FBS, 100 unit/ml of penicillin, 100 μg/ml of streptomycin) on 100 mm petri dishes. Following this incubation step, plates were washed extensively with PBS to remove residual non-adherent red blood cells. The remaining cells were maintained at 37°C/5% CO_2_ in control media. One-week later, when the monolayer of adherent cells reached confluence, cells were trypsinized (0.05% Trypsin-EDTA; Sigma), resuspended in α-MEM containing 10% FBS, and subcultured at a concentration of 2,000 cells/cm^3^. Each subsequent passage via subculture occurred on a weekly basis under similar conditions.

### Colony-forming and cell proliferation assay

To evaluate colony number per inoculated cell, 100 adherent cells at the second passage were re-plated and cultured for seven days on 60 cm^2^ dishes in standard culture medium. The cells were subsequently fixed with 4% paraformaldehyde, stained with 0.5% crystal violet for five minutes, and washed twice with distilled water. The number of colonies was then counted. Colonies less than 2 mm in diameter and faintly stained colonies were not further assessed.

To estimate cell proliferation, adherent cells at this same passage above were plated onto 24-well plates (10^4^ cells/well) in α-MEM containing 10% FBS. MTT (3-(4,5-Dimethylthiazol-2-yl)-2,5-diphenyltetrazolium bromide) assays were performed one, two, three and four days after plating using our established protocol [21]. Briefly, growth medium containing 0.25 mg/ml MTT (Sigma) was added to each well, which was further incubated at 37°C for 20 minutes. After incubation, the MTT solution was removed and 0.2 ml/well dimethyl sulfoxide (DMSO; Sigma) was added to solubilize the cells. The optical absorption of each well was measured at 540 nm with a microtiter ELISA reader (ELX800, BioTek, Winooski, VT, USA).

### Immunophenotypic analysis

Flow cytometric analysis was used to characterize the phenotypes of the MPCs. For experiments, we used the third or fourth passage of MPCs. At least 50,000 cells (in 100 μL PBS, 0.5% bovine serum albumin (BSA), 2 mmol/L EDTA) were incubated with fluorescein isothiocyanate-labeled monoclonal antibodies against human CD90, CD44, CD73, HLA-ABC, CD45, CD31 and HLA-DR (BD Biosciences Clontech, Palo Alto, CA, USA) or with the respective isotype control antibody. After washing, labeled cells were analyzed by flow cytometry using fluorescence-activated cell sorting (FACS) Caliber flow cytometer and Cell Quest Pro software (BD Biosciences, San Diego, CA USA).

### Multilineage differentiation of MPCs

MPCS were analyzed for their capacity to differentiate toward the adipogenic, osteogenic Fand chondrogenic lineages. The third or fourth passage of MPCs was used for these experiments.

Adipogenic differentiation was induced by culturing MPCs for three weeks in adipogenic media (1 μM dexamethasone, 100 μg/ml 3-isobutyl-1 methylxanthine (IBMX), 5 μg/ml insulin and 60 μM indomethacine, and 10% FBS in α-MEM) and assessed using an Oil Red O stain as an indicator of intracellular lipid accumulation. Prior to staining, the cells were fixed for 15 minutes at room temperature in 70% ethanol. The cells were incubated in 2% Oil Red O reagent for one hour at room temperature. Excess stain was removed by washing with 70% ethanol, followed by several changes of distilled water. To quantify adipogenic differentiation, the amount of Oil Red O was determined by measuring the optical density at 510 nm with a spectrophotometer after treatment with isopropyl alcohol. The results were then normalized to the protein contents of the samples.

Osteogenic differentiation was induced by culturing MPCs for three weeks in osteogenic media (0.1 mM dexamethasone, 10 uM β-glycerophosphate, 50 μg/ml ascorbic acid and 10% FBS in α-MEM) and examined for extracellular matrix calcification by Alizarin red S staining. For Alizarin red S staining, the cells were fixed with 70% ethanol and washed with distilled water. The cells were incubated in 2% Alizarin red solution for 15 minutes at room temperature, and washed several times with distilled water to clear the stain. In order to evaluate alkaline phosphate (ALP) activity, an early marker of osteoblast activity, samples were treated with 100 μl 50 mM p-nitrophenyl phosphatase hexahydrate containing 1 nm MgCl_2_, and incubated for 20 minutes at 37°C. To quantify osteogenic differentiation, optical densities were measured at 405 nm with a spectrophotometer. The results were then normalized to the protein content within the samples.

Chondrogenic differentiation was induced using the micromass culture technique. Briefly, 10 μl of a concentrated MSC suspension (3 × 10^5^ cells/ml) were plated into the center of each well and allowed to attach at 37°C for two hours. Chondrogenic media (CM,1% FBS, 0.1 mM dexamethasone (Sigma), 50 μg/ml ascorbic acid, ITS + 1 (insulin-transferrin-selenium; Sigma), 10 ng/ml TGF-β1 (Sigma), 10 ng/ml in α-MEM) was gently overlaid so as not to detach the cell nodules, and cultures were maintained in CM for four weeks prior to analysis. Chondrogenesis was confirmed by immunohistochemistry. Micromasses were harvested after four weeks culture and immediately frozen in optimum cutting temperature medium. Sections 10 μm thick were cut and fixed in 4% paraformaldehyde. For collagen type II staining, sections were first blocked with 10% horse serum, incubated with purified anti-mouse collagen type II antibody (BD Bioscience, San Jose, CA, USA) for one hour, and washed with PBS (pH 7.4). Cells with bound antibodies were detected with a peroxidase substrate kit (Vectastain ABC kit; Vector Laboratories, Burlingame, CA, USA). Sections were washed, counterstained with hematoxylin and examined by light microscopy.

### Quantitative real-time reverse transcription-polymerase chain reaction

For quantitative real-time polymerase chain reaction (qRT-PCR) analysis, total RNA was isolated from each of the cell donor cultures from Day 21 monolayer cultured cells using Trizol reagent (Invitrogen Corp., Carlsbad, CA, USA). Isolated RNA was then reverse-transcribed using random hexamers. RT-PCR was performed using 10 ng of cDNA and SYBR Green mix (Bio-Rad Laboratories, Hercules, CA, USA). Transcript-specific primers were designed based on GenBank cDNA sequences listed in Table 1: (a) signature markers of adipogenesis: lipoprotein lipase (LPL) and peroxisome proliferator-activated receptor-gamma (PPARγ), (b) markers of osteogenesis: ALP and osteocalcin (OC), (c) markers of chondrogenesis: collagen type II α1 (COL2A1) and aggrecan (AGN). Expression levels are presented as the fold increase over that of glyceraldehyde-3-phosphate dehydrogenase (GAPDH), using the formula 2^(ΔCt)^, where ΔCt = Ct of target gene - Ct of GAPDH.

**Table 1:** Reverse transcription-polymerase chain reaction primer sequences

**Primer name**	**Sequence (5′-3′)**	**Size (bp)**
GAPDH	Sense: GGACTCATGACCACAGTCCATGCC	152
	Antisense: TCAGGGATGACCTTGCCCACA	
LPL	Sense: GAGATTTCTCTGTATGGCACC	276
	Antisense: CTGCAAATGAGACACTTTCTC	
PPARγ	Sense: TGAATGTGAAGCCCATTGAA	161
	Antisense: CTGCAGTAGCTGCACGTGTT	
ALP	Sense: TGGAGCTTCAGAAGCTCAACACCA	454
	Antisense: ATCTCGTTGTCTGAGTACCAGTCC	
OC	Sense: ATGAGAGCCCTCACACTCCTC	294
	Antisense: GCCGTAGAAGCGCCGATAGGC	
AGN	Sense: TGCGGGTCAACAGTGCCTATC	182
	Antisense: CACGATGCCTTTCACCACGAC	
COL2A1	Sense: GGAAACTTTGCTGCCCAGATG	167
	Antisense: TCACCAGGTTCACCAGGATTGC	

AGN, aggrecan; ALP, alkaline phosphatase; bp, base pairs; COL2A1, collagen type II α1; GAPDH, glyceraldehydes-3-phosphate dehydrogenase; LPL, lipoprotein lipase; OC, osteocalcin; PPARγ, peroxisome proliferator-activated receptor-gamma.

### Detection of cytokine secretion by MPCs

Secretion of interleukin (IL)-2, IL-4, IL-5, IL-6, IL-8, IL-10, IL-13, tumor necrosis factor (TNF)-α, interferon (INF)-γ, transforming growth factor (TGF)-β was detected by DuoSet Human Immunoassay (R&D Systems, Wiesbaden, Germany), according to the manufacturer’s protocol. Third-passage cells were washed three times with Dulbecco’s modified Eagle’s medium (DMEM) and cultured for 48 hours in standard medium at a concentration of 1 × 10^6^ cells/ml. To evaluate the responsiveness of MPCs to immunological signals, cells were incubated with TNF-α (1,000 U/ml) and IFN-γ (400 U/ml) for 16 hours. Supernatant samples were run in triplicate and compared to standard curves.

### Statistical analysis

All experiments were repeated at least in triplicate. Data are presented as mean ± SEM from all tissue specimens isolated. Statistical significance was assessed by one-way repeated analysis of variance (ANOVA), followed by Tukey’s honest significant difference (HSD) *post hoc* test using SPSS software package version 13.0 (SPSS Inc., Chicago, IL, USA). A value of *P* <0.05 was considered significant.

## Results

### Isolation and proliferation characteristics of MS-MPCs, ES-MPCs, IT-MPCs and T-MPCs

**Figure 1:**
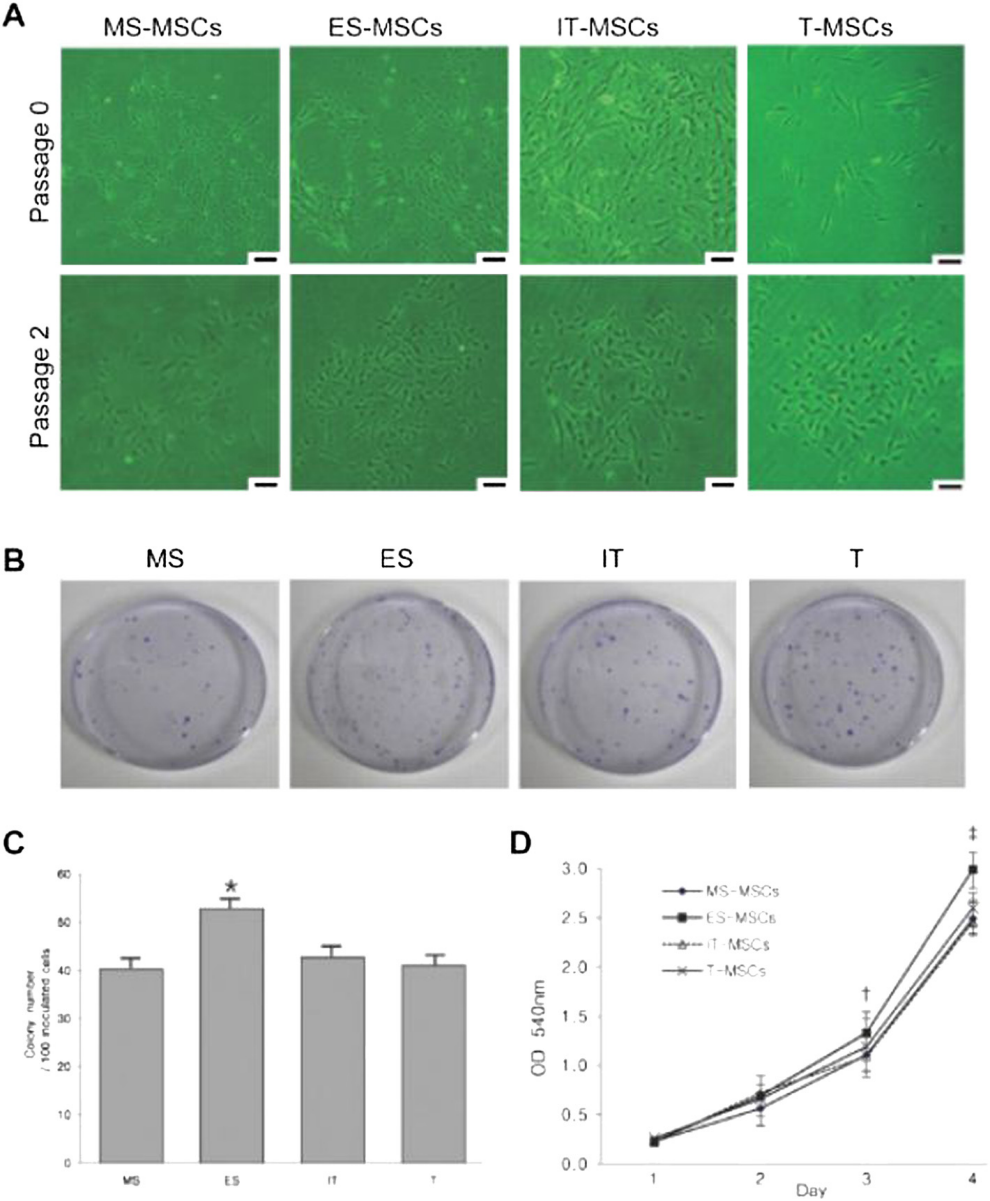
**Isolation characteristics and proliferation capability. (A)** The fibroblast-like morphology of MPCs is shown after initial plating and at passage 2 (original magnification 100×, scale bar = 30 μm). **(B)** A total of 100 adherent cells were cultured in 60 cm^2^ dishes for seven days and stained with 0.5% crystal violet. **(C)** The colony number per adherent cells was significantly higher in ES than IT, MS and T. **(D)** ES-MPCs proliferated at a faster rate compared with MS-, IT and T-MPCs at days 3 and 4 by MTT assay. Data are expressed as the mean ± SEM. * *P =* 0.004, †*P* = 0.001, ‡ *P <*0.001. ES, ethmoid sinus; IT, inferior turbinate; MPCs, mesenchymal progenitor cells; MS, maxillary sinus; T, tonsil.

At an initial plating density of 1 × 10^6^ cells/cm^2^, MS, ES, IT and T-derived fibroblastoid cells formed a monolayer four to five days after initial plating. These putative MS, ES, IT and T-derived MPCs (IT, MS, ES and T-MPCs) were of serpiginous or fibroblast-like morphology similar to previously reported adipose tissue and bone marrow-derived MPCs (Figure 1A). This morphology was maintained during passage in monolayer culture.

To examine the frequency of MPCs present from each cell source, we observed the colony-forming capabilities of each MSC type and counted the number of colonies in dishes stained with crystal violet (Figure 1B). The number of colonies per 100 inoculated cells, that is, colony-forming efficiency, was significantly higher in ES than MS, IT and T tissues (*P* = 0.004). However, no significant differences were found among the MS, IT and T groups (Figure 1C).

The proliferation capability of MS-MPCs, ES-MPCs, IT-MPCs and T-MPCs was analyzed by MTT assay. Although plated at the same initial cell number (1 × 10^4^ cells/well), ES-MPCs proliferated at a faster rate compared with MS-MPCs, IT-MPCs and T-MPCs at days 3 and 4 (*P* = 0.001 and *P <*0.001, respectively) (Figure 1D). On days 1 and 2, no significant differences were noted between any of the groups.

### Immunophenotypic characterization

**Figure 2:**
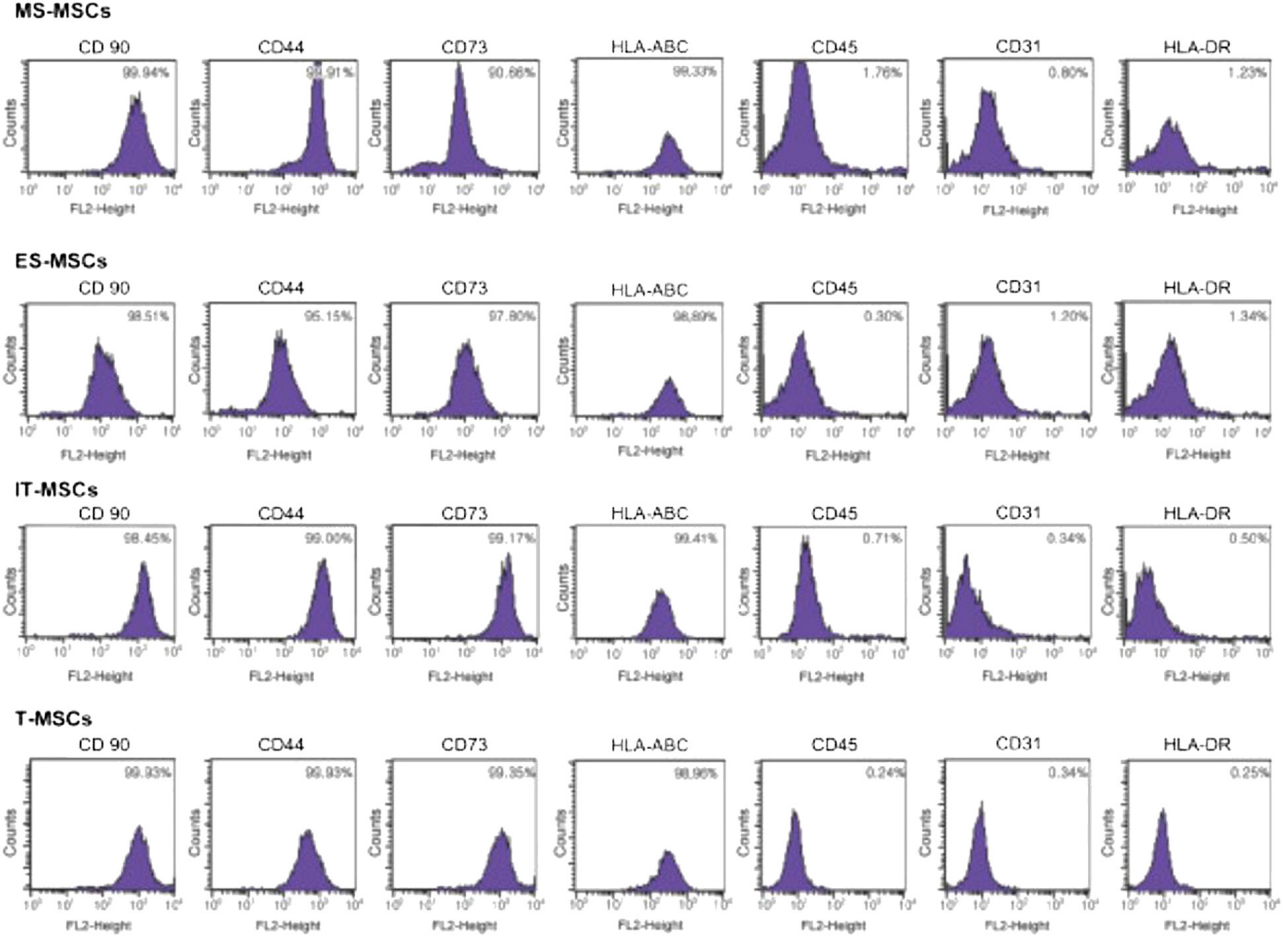
**Immunophenotypic analysis of MS-, ES, IT- and T-MPCs.** Flow cytometry showed positive expression for CD90, CD44, CD73 and HLA-ABC and negative expression for CD45, CD31 and HLA-DR. The values represent the mean percentage of all assessed cells positively stained by the respective antibodies. ES, ethmoid sinus; IT, inferior turbinate; MPCs, mesenchymal progenitor cells; MS, maxillary sinus; T, tonsil.

To further characterize these cells, cell surface markers were examined by flow cytometry. MPCs from all sources were positive for CD90, CD44, CD73, and HLA-ABC and negative for CD45, CD31, and HLA-DR, with no significant differences noted between the four cell source populations (Figure 2).

### Adipogenic differentiation capacity

**Figure 3:**
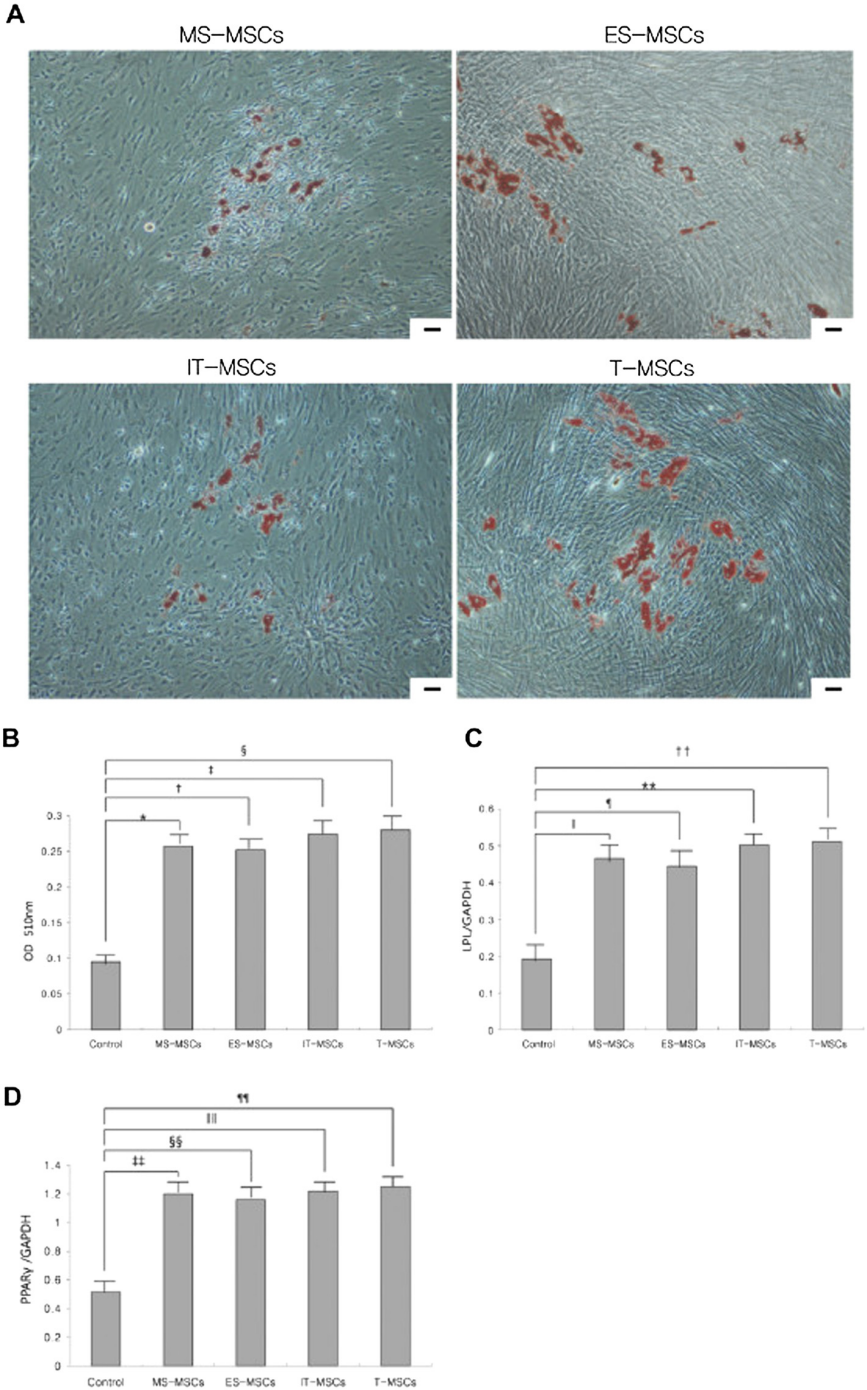
**Comparative analysis of adipogenic differentiation capacity of MS-, ES, IT- and T-MPCs. (A)** Adipogenesis was detected by the formation of multiple, intracellular lipid-filled droplets stained with Oil Red O after induction for 21 days (original magnification 100×, scale bar = 50 μm). **(B)** In the spectrophotometric analysis of Oil Red O staining, optical densities were significantly higher in MS-, ES-, IT- and T-MPCs groups than the control group. No significant differences were found between any of the MPCs groups. The expression of specific adipogenic genes was evaluated by real-time qRT-PCR. LPL **(C)** and PPARγ **(D)** were up-regulated during adipogenesis in all MPCs groups. However, there were no significant differences in the expression level of two markers among any of the MPCs groups. Data are expressed as the mean ± SEM. *, ‡ *P =* 0.003, †, ‡‡ *P* = 0.002, § *P* = 0.017, ǁ *P* = 0.005, ¶, ** *P <*0.001, †† *P* = 0.010, §§, ǁǁ *P* = 0.002, ¶¶ *P* = 0.020*.* ES, ethmoid sinus; IT, inferior turbinate; LPL, lipoprotein lipase; MPCs, mesenchymal progenitor cells; MS, maxillary sinus; PPARγ, peroxisome proliferator-activated receptor-gamma; T, tonsil.

Adipogenic differentiation was demonstrated by the accumulation of neutral lipid vacuoles indicated by the Oil Red O staining. After adipogenic induction, a significant fraction of the cells contained multiple, intracellular lipid-filled droplets that stained by Oil Red O in MS-, ES-, IT- and T-MPCs (Figure 3A). No red staining was detected in control groups (data not shown). Optical densities were significantly higher in MS-, ES-, IT- and T-MPCs groups than in the control group (*P* = 0.003, *P* = 0.002, *P* = 0.003 and *P* = 0.017, respectively). However, there were no significant differences among any of the MPCs groups (Figure 3B).

The expression of LPL and PPARγ were analyzed by qRT-PCR after 21 days of induction. The expression of LPL and PPARγ were both up-regulated during adipogenesis in all MPCs groups. However, there were no significant differences in the expression level of two markers among any of the MPCs groups (Figure 3C,D).

### Osteogenic differentiation capacity

**Figure 4:**
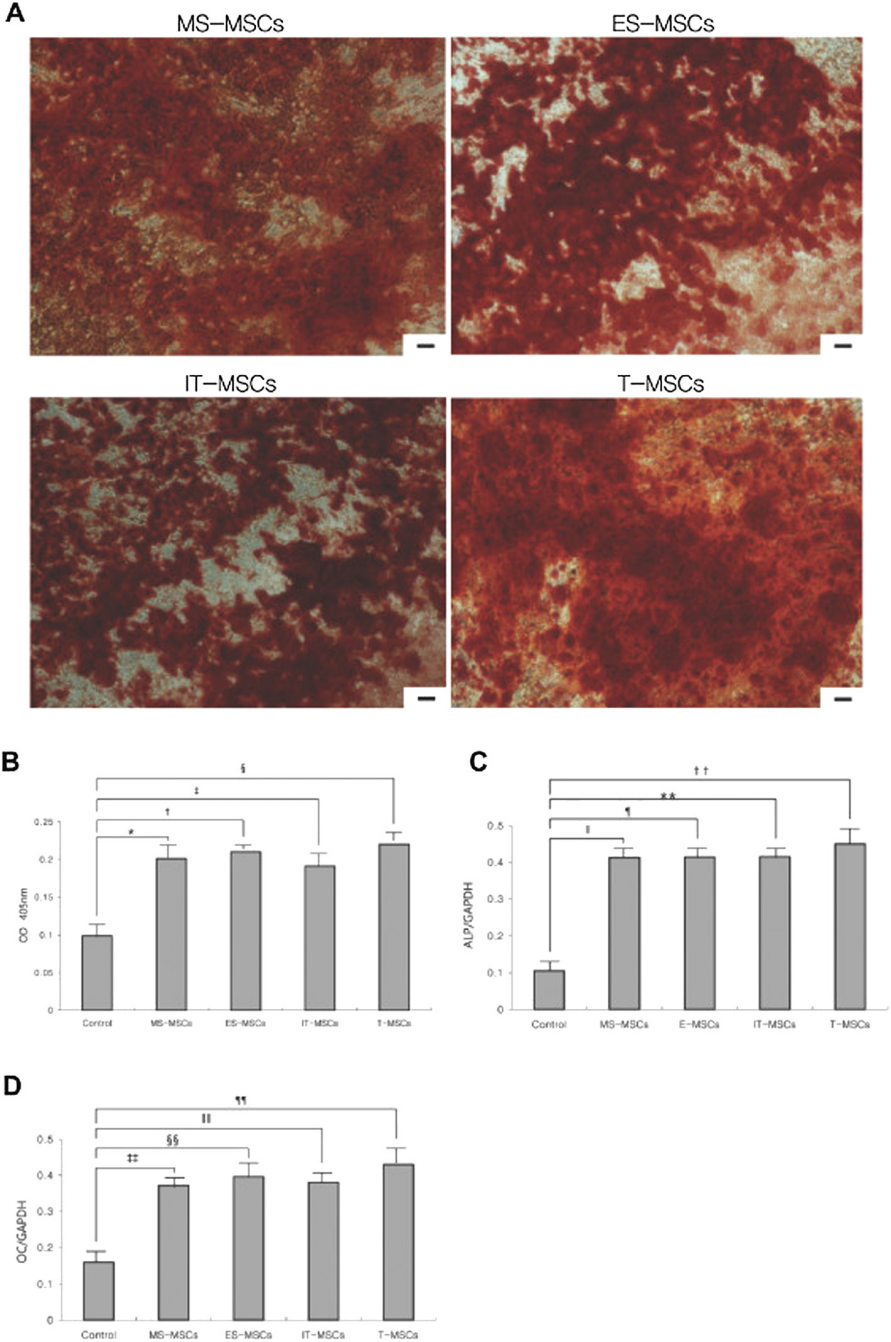
**Comparative analysis of osteogenic differentiation capacity of MS-, ES, IT- and T-MPCs. (A)** Osteogenesis was demonstrated by several dark red regions stained with Alizarin Red S, indicative of calcified extracellular matrix under osteogenic differentiation condition (original magnification 100×, scale bar = 50 μm). **(B)** In the analysis of alkaline phosphatase activity, optical densities were significantly higher in MS-, ES, IT- and T-MPCs groups than control group. However, there were no significant differences among any of the MPCs groups. Up-regulation of the expression of specific osteogenic genes, *ALP***(C)** and *OC***(D)**, were evaluated by real-time qRT-PCR. Data are expressed as the mean ± SEM. *, †, ‡, ǁ, ¶, **, ‡‡, §§, ǁǁ *P <*0.001, § *P* = 0.006, †† *P* = 0.042, ¶¶ *P* = 0.040. *ALP*, alkaline phosphate; ES, ethmoid sinus; IT, inferior turbinate; MPCs, mesenchymal progenitor cells; MS, maxillary sinus; *OC*, osteocalcin; T, tonsil.

Osteogenic differentiation was confirmed by the detection of an osteogenic phenotype consisting of an increased expression of ALP and the deposition of Alizarin red S stained mineralized matrix, calcification appearing as red regions within the cell monolayer. Consistent with osteogenesis, several red regions in Alizarin red S staining were observed in MS-, ES-, IT- and T-MPCs (Figure 4A). No red regions were detected in control groups (data not shown). ALP activities were significantly higher in MS-, ES-, IT- and T-MPCs groups than in the control group (*P <*0.001, *P* <0.001, *P <*0.001 and *P* = 0.006*,* respectively). However, there were no significant differences among any of the MPCs groups (Figure 4B).

The expression of osteogenic genes was assessed by qRT-PCR. Up-regulated mRNA expression of *ALP* and *OC* was observed in all MPCs groups. No significant differences in the expression level of these genes were seen among any of the MPCs groups (Figure 4C,D).

### Chondrogenic differentiation capacity

**Figure 5:**
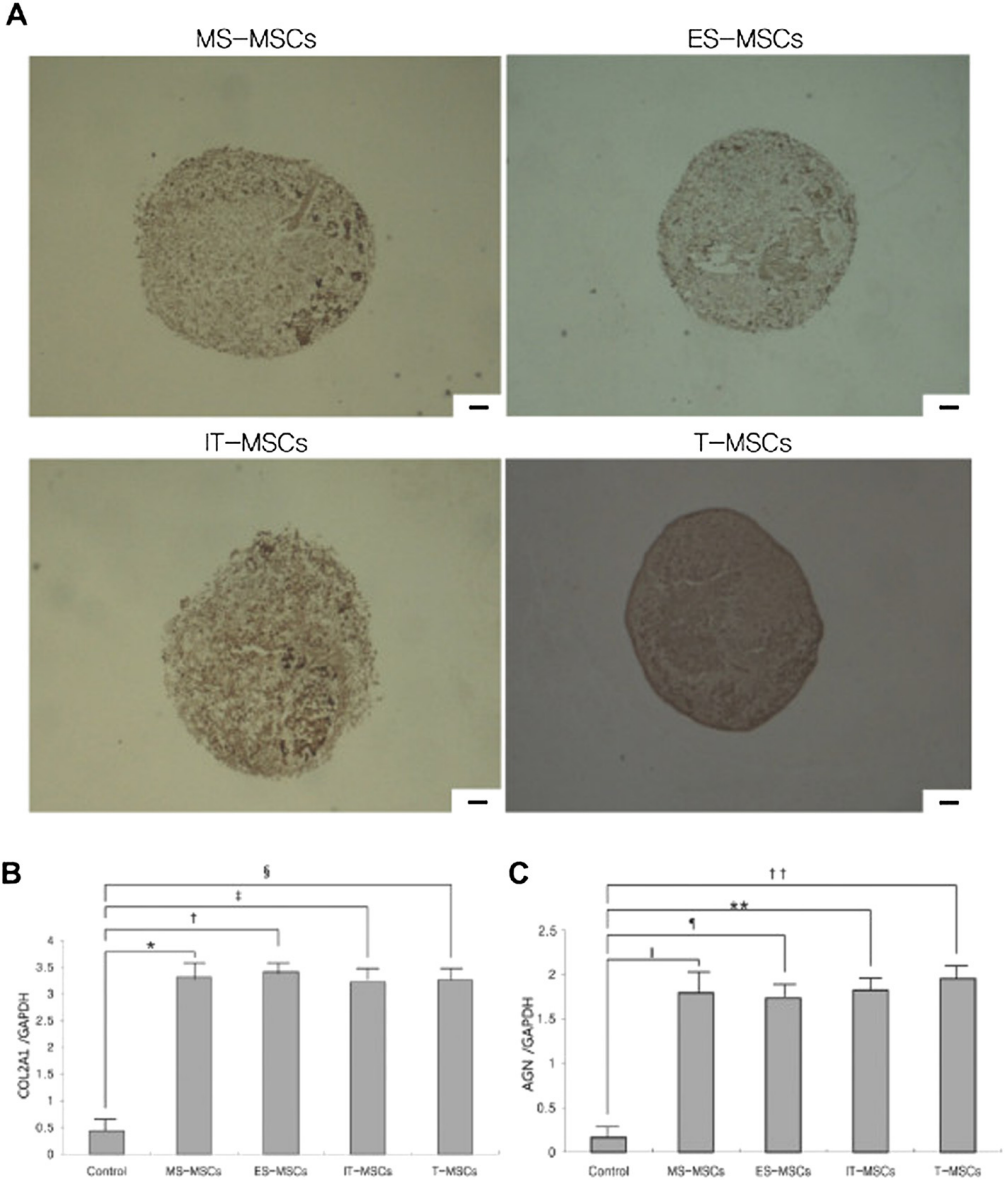
**Comparative analysis of chondrogenic differentiation capacity of MS-, ES, IT- and T-MPCs. (A)** Chondrogenesis was demonstrated by the formation of a sphere and type II collagen expression. There were no differences in the size of sphere and the immunohistochemical staining for type II collagen among any of the MPCs groups (original magnification 100×, scale bar = 50 μm). The expression of specific chondrogenic genes, *COL2A1***(B)** and *AGN***(C)**, was evaluated by real-time qRT-PCR. *COL2A1* and *AGN* were up-regulated during chondrogenesis in all MPCs groups. However, there were no significant differences in the expression level of two markers among any of the MPCs groups. Data are expressed as the mean ± SEM. *, †, ‡, ǁ, ¶, ** *P <*0.001, § *P* = 0.002, †† *P* = 0.005. AGN, aggrecan; COL2A1, collagen type II α1; ES, ethmoid sinus; IT, inferior turbinate; MPCs, mesenchymal progenitor cells; MS, maxillary sinus; T, tonsil.

Compared with the control group, chondrogenic differentiation of MPCs was confirmed by the formation of a sphere in micromass culture. The size of a sphere was not different among the MPCs groups (Figure 5A). Chondrogenesis was further studied by analyzing the expression of cartilage-specific type II collagen. There was no significant difference in the expression of collagen type II among any of the MPCs groups.

*COL2A1* and *AGN* mRNA levels increased in the MS-, ES-, IT- and T-MPCs groups. However, there were no significant differences in the expression level of these genes between the MPCs groups (Figure 5B,C).

### Analysis of cytokine secretion in MPCs

**Figure 6:**
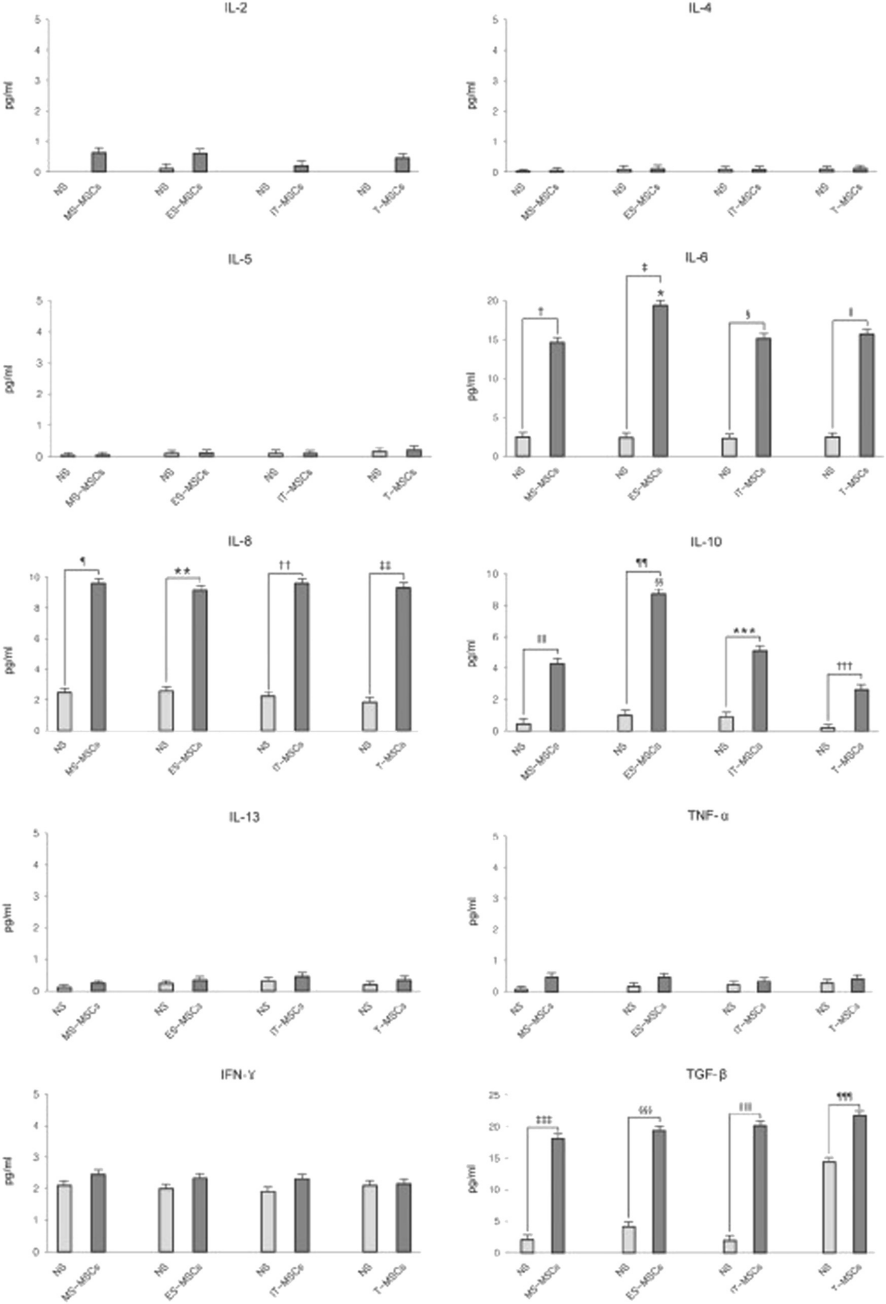
**Cytokine secretion in MS-, ES, IT- and T-MPCs.** IL-6, IL-8, IFN-γ and TGF-β were constitutively secreted and easily exceeded 1 pg/ml under standard culture conditions. However, IL-2, IL-4, IL-5, IL-10, IL-13 and TNF-α were secreted less than 1 pg/ml or not detected. MS-, ES-, IT- and T-MPCs upon stimulation secreted a large amount of IL-6, IL-8, IL-10 and TGF-β. The expression of IL-6 and IL-10 were significantly higher in the ES-MPCs than MS-, IT- and T-MPCs. Data are expressed as the mean ± SEM. *, ǁ, ¶, **, ‡‡‡, §§§, ǁǁǁ *P* = 0.001, † *P* = 0.009, ‡, §§, ¶¶, ¶¶¶ *P <*0.001, § *P* = 0.006, †† *P* = 0.003, ‡‡ *P* = 0.004, ǁǁ *P* = 0.006, *** *P* = 0.042, ††† *P* = 0.046. ES, ethmoid sinus; IT, inferior turbinate; MPCs, mesenchymal progenitor cells; MS, maxillary sinus; T, tonsil.

To test whether MPCs display immune cell-like features, secretion of cytokines and response to inflammatory signals were analyzed. MS-, ES-, IT- and T-MPCs constitutively secreted IL-6, IL-8, IFN-γ and TGF- β that easily exceeded 1 pg/ml under standard culture conditions, although IL-2, IL-4, IL-5, IL-10, IL-13 and TNF-α were secreted less than 1 pg/ml or not detected (Figure 6). Furthermore, we investigated the responsiveness of MPCs to immunological signals and exposed these cells to a panel of cytokines, including TNF-α and IFN-γ. MS-, ES-, IT- and T-MPCs upon exogenous stimulation secreted much larger amounts of IL-6, IL-8, IL-10 and TGF-β. Interestingly, the expression of IL-6 and IL-10 were significantly higher in the ES-MPCs than MS-MPCs, IT-MPCs and T-MPCs (*P* = 0.001 and *P <*0.001, respectively).

## Discussion

MPCs were initially isolated from bone marrow but are now shown to reside in almost all adult organs and tissues [22]. The characteristics of MPCs depend not only on species-specific factors but also on the tissue sources from which the MPCs were harvested. Furthermore, the isolation of MPCs from new tissue sources is critical as currently defined donor tissues have specific disadvantages, including large donor site morbidity as well as a dearth of MPCs upon harvest, requiring generous amounts of donor tissue to be harvested.

Ideal progenitor cell populations would be autologous cells that can be harvested without difficulty from each patient, manipulated efficiently *in vitro*, and autoimplanted back into the same patient [23]. In this study, we focused on the sinus mucosa, which is an easily accessible donor tissue site that is routinely acquired as part of endoscopic sinus surgery, an exceedingly commonly performed surgical procedure throughout the world. Additionally, harvest of these tissues is ethically acceptable, since healthy ‘bystander’ mucosal specimens that are acquired during surgery are routinely discarded. In this report, we isolated and cultured MPCs from the mucosa of maxillary and ethmoid sinuses, and mesenchymal-like characteristics were able to be identified in both MS-MPCs and ES-MPCs, such as surface marker expression, plastic adherence, self-renewal and multilineage differentiation capacity. In addition, we compared these two MPCs to IT-MPCs and T-MPCs in the same individuals. The latter cells were recently reported as an alternative source of MPCs in order to choose more optimal cell source for particular therapeutic purposes.

The respiratory mucosa of maxillary and ethmoid sinus is composed of a superficial epithelial lining and the underlying submucosa/lamina propria. The outer surface is a ciliated pseudostratified epithelium, while the deeper portion underneath a basement membrane is a highly vascularized connective tissue. Although it is difficult to determine the precise location of MPCs within the sinus mucosa, a number of studies have suggested that MPCs are likely to be localized within the lamina propria underlying the basement membrane [24-26].

The morphological features of MS-MPCs, ES-MPCs, IT-MPCs and T-MPCs were similar to those described for BM-MPCs and showed plastic adherence and fibroblast-like growth. After an initial lag phase for four to five days, these adherent cells proliferated actively and became nearly confluent. Although adherent cells give rise to a mixed population of cells, only progenitor cells would possess the properties of self-renewal and multilineage potential. To identify colony-forming MPCs and to determine their proliferation capacity, the colony-forming efficiency and MTT assays were evaluated. Among the four MPCs derived from MS, ES, IT and tonsil, we unexpectedly discovered that ES-MPCs have the highest colony-forming potential and proliferation capability in culture. Furthermore, we demonstrate that ES-MPCs may potentially have higher proliferation capacity than BM-MPCs as previous studies have revealed that T-MPCs possess faster population doubling time than BM-MPCs [16].

Several previous reports have documented the expression of phenotypic markers in MPCs [27-29]. Immunophenotypic analysis of this study showed that the surface marker profiles of MS-, ES-, IT- and T-MPCs were compatible with those previously reported for MPCs. These cells were positive for CD90, CD44 and CD73 but negative for CD45, CD31 and HLA-DR.

Multilineage differentiation potential has been considered an important quality of MPCs. As shown by histochemical or immunohistochemical staining, optical density and qRT-PCR for defined lineage-specific marker genes, MS-, ES-, IT- and T-MPCs shared the capacity to differentiate into adipogenic, osteogenic and chondrogenic lineages in the presence of tissue-specific induction medium. However, there were no significant differences in multilineage differentiation capacity among any of the MPC groups.

MPCs have recently been demonstrated to suppress T-, B-, natural killer (NK) and dendritic cell activities, thus exerting an immunoregulatory influence both *in vitro* and *in vivo*[5,30-33]. Although the immunosuppressive mechanisms of MPCs remain to be clarified, it is generally accepted that the ability of MPCs to modulate immune responses relies on cell contact-dependent mechanisms and select soluble factors secreted by MPCs. Several soluble factors have been proposed to mediate the immunosuppressive effect, including IL-6, IL-8, IL-10, IFN-γ, TGF-β, granulocyte-colony stimulating factor (G-CSF) and granulocyte macrophage-colony stimulating factor (GM-CSF) [23,34]. In the present study, all of MSC populations studied constitutively, or upon stimulation, secrete large amounts of IL-6, IL-8, IL-10, IFN-γ and TGF-β. Interestingly, after stimulation with TNF-α and IFN-γ, we detected significantly higher secretions of IL-6 and IL-10 by ES-MPCs. IL-6 has been known to stimulate antibody secretion in human B cells [35] and IL-10 has been recognized to inhibit the differentiation of dendritic cells and increase the number of regulatory T cells [23]. Taken together, these data show that ES-MPCs may be a highly robust source for MSC-mediated immunomodulation.

Our studies also revealed similarities and differences among four different sources of MPCs from the same individual. MS-, ES-, IT and T-MPCs can be regarded as potential cell sources for possible tissue engineering, regenerative medicine and immunomodulatory strategies. It is thought that clinical applications of MPCs may be based not only on their differentiation potential, but more likely on the abundance, frequency, proliferation potential and immunosuppressive capacity. In this regard, ES-MPCs may be an ideal source of MPCs in respect to easily repeatable accessibility without donor site morbidity, highest proliferation ability and elevated secretion of soluble factors. These differences in properties of MPCs isolated from the ES and MS might be explained by differences in embryological development, as the endochondral origin of the ES makes these regions ontologically different from the other paranasal sinuses [36].

Human ethmoid sinus mucosa can be easily obtained via transnasal endoscopic approach without incision under topical anesthesia at outpatient clinics. Respiratory epithelium appears to regenerate mostly from undifferentiated basal cells from adjacent non-injured areas. If we preserve the mucoperiosteum over the bone, and thereby avoid areas of bone exposure, mucosal dysfunction can be reduced after removal of sinus mucosa. Especially, the ethmoid bulla is typically a rather large anterior ethmoid air cell and the most consistent and recognizable of the ethmoid cells. Most of the ethmoid sinus mucosa can be easily obtained from the inferior and medial portion of the ethmoid bulla. Therefore, major complications, such as a cerebrospinal fluid leak or orbital hemorrhage, rarely develop.

This study is not devoid of inherent limitations. This *in vitro* study does not necessarily translate into MPCs that can or will exhibit the same potential *in vivo*. Another consideration for the future clinical use of MPCs and notably ES-MPCs, is that these cells may be isolated from sinus mucosa that are frequently inflamed and infused with inflammatory mediators, and, therefore, individual differences in the patient mucosa due to local tissue effects may affect the properties of the MPCs. Future studies will address the exact characteristics and localization of sinus-derived MPCs and elucidate the effects of inflammatory disease on MPCs both *in vitro* and *in vivo*.

## Conclusions

This study demonstrates that MPCs with multilineage differentiation potential and immunosuppressive property can be obtained from MS and ES mucosa. Most notably, ES-MPCs could be a novel and promising abundant source of MPCs due to their high proliferation ability and superior capacity toward secretion of immunomodulatory cytokines.

## Abbreviations

α-MEM: α-modified Eagle’s medium; AGN: Aggrecan; ALP: Alkaline phosphatase; BM: Bone marrow; COL2A1: Collagen type II α1; ES: Ethmoid sinus; FBS: Fetal bovine serum; GAPDH: Glyceraldehydes-3-phosphate dehydrogenase; IL: Interleukin; INF: Interferon; IT: Inferior turbinate; LPL: Lipoprotein lipase; MPCs: Mesenchymal progenitor cells; MS: Maxillary sinus; MTT: 3-(4,5-Dimethylthiazol-2-yl)-2,5-diphenyltetrazolium bromide; OC: Osteocalcin; PBS: Phosphate-buffered saline; PPARγ: Peroxisome proliferator-activated receptor-gamma; qRT-PCR: Quantitative real-time polymerase chain reaction; T: Tonsil; TGF: Transforming growth factor; TNF: Tumor necrosis factor.

## Competing interests

The authors declare that they have no competing interests.

## Authors’ contributions

KSC participated in drafting the manuscript, acquisition of data, analysis and interpretation of data, and the conception and design of the work. HYP participated in acquisition of data, and analysis and interpretation of data. HJR participated in the interpretation of data. DB participated in the acquisition of data. PH participated in acquisition and interpretation of data. JN participated in the conception and design of the work, interpretation of data and editing of the manuscript. All authors read and approved the final manuscript.
